# IHMC’s experience competing in the Cybathlon compared to the DARPA robotics challenge

**DOI:** 10.1186/s12984-017-0324-0

**Published:** 2017-11-09

**Authors:** Peter Neuhaus

**Affiliations:** IHMC, 40 S. Alcaniz St, Pensacola, FL 32502 USA

**Keywords:** Cybathlon, Exoskeleton, Competition, DRC

## Abstract

**Background:**

As a research scientist, my work tends to focus on scientific investigations. Our group occasionally makes discoveries or has a successful demonstration, and sometimes we can even repeatedly demonstrate something working on the hardware. This mode of operation works for research, but not for competitions. In the past few years, I have participated in two international robotics competitions, the DARPA Robotics Challenge (DRC) and the Cybathlon; the research and development process for these competitions is significantly different from our typical research work. This commentary discusses our experience preparing for the Cybathlon, and contrasts it with our experience with the DRC.

**Main body:**

The human in the loop for the Cybathlon was the biggest differentiator between the DRC and the Cybathlon. Having the human at the center of the competition not only changed the way we developed, but changed how we viewed the impact of our work. For the DRC, a physics based dynamic simulation was a powerful, and invaluable, tool for not only the algorithm developers, but the robot operator as well. For the Cybathlon, simulation was of little use because the all of closed-loop control was performed by the pilot. In the software development cycle for the Cybathlon, the push was to just come up with something that works and “lock it down” and do not change it, so that the pilot could train with a given set of motions that would not change and make up for any deficiencies with his own abilities. The Cybathlon was more of an athletic challenge for the human who was assisted by technology. The DRC was the opposite, it was a robotics challenge assisted by a human. This commentary focuses on describing the Florida Institute for Human and Machine Cognition’s (IHMC) experience leading up to and at the Cybathlon, with some comparisons to the DRC experience.

**Conclusion:**

The Cybathlon was a very worthwhile experience me, my team, and of course our pilot. Knowing that our development could improve the quality of life and health for a group of people was very motivating and rewarding. Engineering competitions accelerate development, engage the public, and in the case of the Cybathlon, increase public awareness of issues for people with disabilities. The Cybathlon also revealed that the powered exoskeleton technology is still nascent in its ability to be a viable alternative to the wheelchair. But with continued developments toward the 2020 Cybathlon, we hope the capabilities of these devices can offer will be significantly improved.

## Background

Competitions are a great way to accelerate performance and engage the public. When we think of spectator competitions, what generally comes to mind first are athletic ones, but competitions in engineering, and more specifically robotics, have recently gained ground in their widespread popularity. MIT has been using design competitions in its mechanical engineering program for over three decades, and recently robotics competitions have become very popular with high school students. As a research scientist, most of my work has been in conducting basic science investigations. However, in the past few years, I have had the opportunity to participate in two professional level robotics competitions: the Cybathlon Powered Exoskeleton Race [1] (October 2016) and the DARPA Robotics Challenge (DRC) Finals [2] (June 2015).

The Cybathlon is a championship for people with disabilities competing in six disciplines, using advanced assistive devices. The Power Exoskeleton Race, one of the six, requires paralyzed athletes to complete six challenges based on common, everyday tasks, in a race against the clock and the competitors. The six tasks are sitting down on a sofa and standing up; walking a slalom course; walking up a steep ramp, opening a door and walking through it, and walking down a steep ramp; walking over stepping stones; walking on tilted surfaces; and walking up and down stairs.

There are several strong similarities between the DRC and the Cybathlon. Both were open to entrants worldwide, creating a truly global competition. Both competitions required the competitor to complete a series of tasks (8 for the DRC, and 6 for the Cybathlon) with the focus foremost on completion with a secondary emphasis on time. The tasks were designed to represent real world challenges that the competitors would face; in the case of the DRC, it was a disaster situation, and for the Cybathlon, it was mobility challenges of everyday life. And while both competitions involved a human, it was in very distinctly different ways.

The Cybathlon is focused around the athletes, who are required to have a given disability, and how they are assisted by technology (the robot). The DRC centered around the robot itself, which was controlled by human operators. In each of these competitions, our success can be directly attributed to the skill of the human. For the DRC, one of our team members was by far the best robot operator at IHMC, and his video game playing skills led to our success. In the case of our Cybathlon pilot, it was his balance, strength, and agility that helped us succeed. Mark Daniel, our Cybathlon pilot, who assisted us in evaluating our previous two exoskeletons over the past six years, was available full time to work with us in the six months leading up to the competition.

This commentary mainly provides a recount of IHMC’s experience preparing for and competing in the Cybathlon. But I also have the unique experience of being part of DRC as well, and part of this commentary is devoted to comparing these two pioneering technology events.

## Main text

For the Cybathlon, we developed our own robot hardware. We were motivated to do this primarily for two reasons; the first is that there are no commercially available exoskeletons that can be purchased for this purpose. In the United States, these types of exoskeletons are considered medical devices, and thus are regulated by the Food and Drug Administration (FDA). The three devices in the US that do have FDA approval are the Ekso from Ekso Bionics, the ReWalk from ReWalk Robotics, and the Indego, from Parker Hannifin Corporation. All three devices have almost identical approval from the FDA to perform ambulatory functions in a rehabilitation institution, and none of the devices are intended for sports or stair climbing. Even if we could purchase one of these devices, they do not offer the functionality that we need and it would be unlikely that they would allow us to alter the software and use the device for an unapproved activity.

As for research devices that might be available, the majority of effort in the United State in mobility assistance for people with paralysis has been focused on the commercial developed of the products by Ekso, ReWalk, and Parker Hannifin. Some research in this area is still being conducted by Prof. Kazerooni (founder of Ekso Bionics) at the University of California, Berkeley, and Prof. Goldfarb (founder of the Indego) at Vanderbilt University. Internationally, some of the leaders in the field are a group at ETH Zurich, EPFL in Switzerland, SG Mechatronics from South Korea, and Roki Robotics from Mexico. But we felt the best, and only, hardware option was to design and build our own device.

Designed as our entry to the 2016 Cybathlon, Mina v2 is the latest exoskeleton developed by IHMC. The main hardware and software development occurred in the 9 months prior to the competition. The team consisted of about eight people, most of whom had just joined IHMC. The team consisted of two mechanical engineers, one electrical, three software, and one embedded programmer. We consulted with an orthotist for help with the design and fit of the leg cuffs and the body interface.

This design drew on our experience with the design and manufacture of Mina v1 [3], the NASA X1 exoskeleton [4] and the Hopper exercise exoskeleton [5]. Mina v2 features a fully custom, carbon composite design. The device includes six electric actuators, which are integrated into the structure as load bearing components, and a protective backpack for electronics. The exoskeleton also features sagittal plane actuators at the hips, knee, similar to all of the other Cybathlon competitors. However, from our work with these devices and with our humanoid robotics work, we know the importance of the ankle in taking large steps, walking quickly, and performing active balance control, therefore it also includes an actuator at the ankle, which none of the other exoskeletons have. We believe that this inclusion of this ankle actuator was a major factor in our success.

Mina v2 functions as a prototype device, designed and built to custom dimensions specifically to fit our pilot. Future modifications will include adjustable links to fit other pilots, the design of which were not feasible within the time constraints of this project.

The actuators themselves are custom Linear Linkage Actuators (LLA), which are modular in construction, allowing for ease of replacement, accessibility, and repair. They were designed in-house, specifically for use with Mina v2, and feature a frameless electric motor, integrated electronics, and an onboard motor amplifier and controller for distributed joint-level control.

Other than the motor controllers, all other electrical components are housed in the 7.5 kg backpack. Central control is performed on an embedded computer. The embedded computer communicates with the motor drivers and other distributed sensors over EtherCAT, an Ethernet-based protocol ideal for hard real-time automation requirements.

**Fig. 1:**
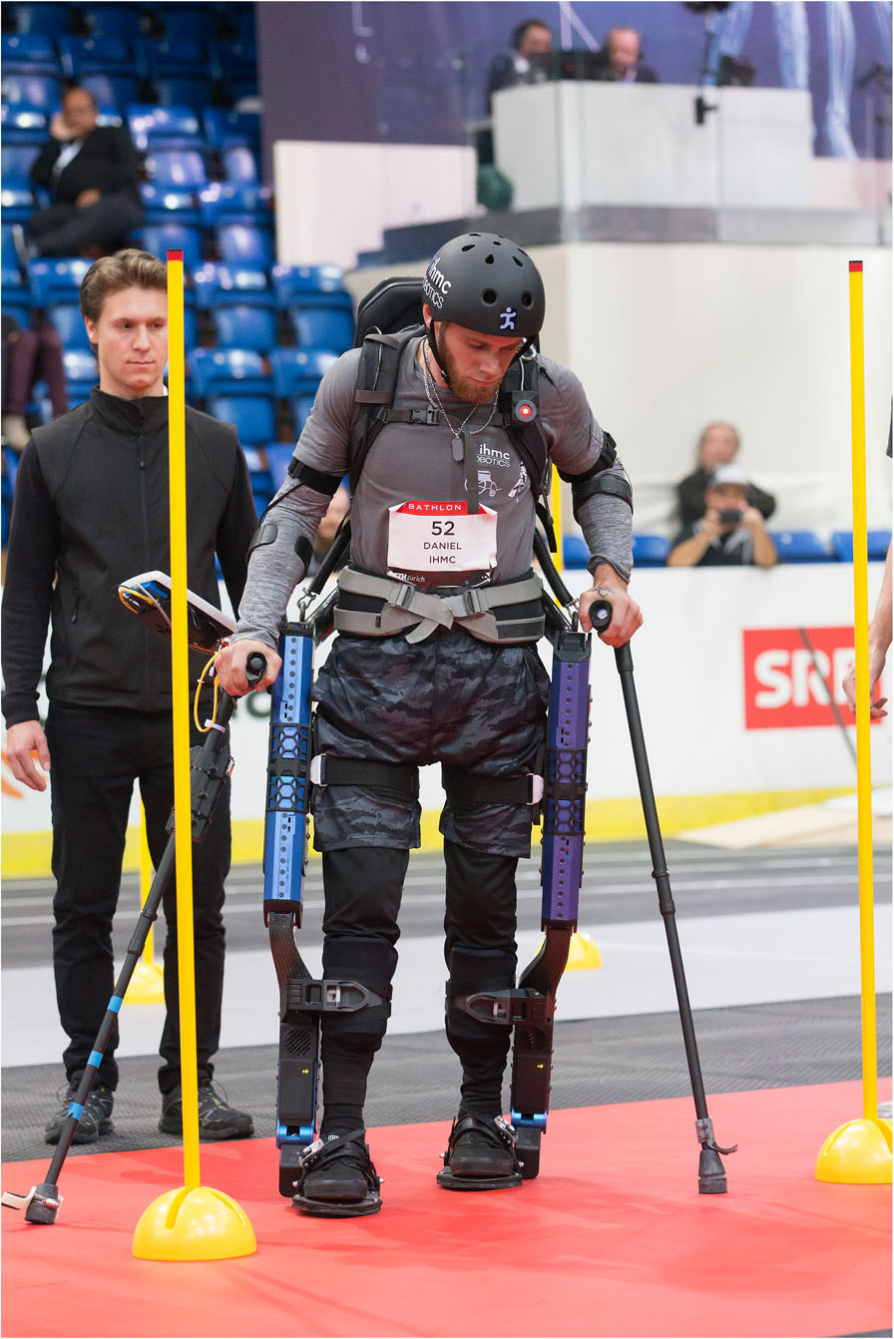
Mark Daniel, the pilot for the IHMC team, competing at the 2016 Cybathlon Powered Exoskeleton Race (ETH Zurich / Nicola Pitaro)

Mina v2 is powered by a 48 V, 480 Wh Lithium Ion battery designed for electric bicycles, and is capable of approximately 2.5 h of fully powered autonomous runtime. Including the 2.3 kg battery, the total exoskeleton mass is 34 kg. The exoskeleton supports its own weight with a load path to ground, so user does not feel any of this weight (Fig. 1).

Designing and building our own hardware ended up taking much longer than we had planned, which resulted in less time for software development and training for Mark. Whereas with the DRC, we could develop our software algorithms without the hardware by utilizing our simulation software. Our DRC robot operator could even train without the hardware by utilizing our simulation. With the Cybathlon, however, much of the preparation for the competition involved having the pilot train in the device and tuning the gait parameters in real time based on his feedback. Think of a cyclist trying to prepare for a bicycle race with only very little time on a bicycle. With our hardware complete, our pilot took his first steps in the exoskeleton eight weeks before the competition. Prior to this, our pilot had about 20 h in our previous two devices over the past six years.

With only eight weeks until we had to pack up and a lot left to do, we had to triage our development, “tossing overboard” any development that was not on the critical path for succeeding in the competition. Being a researcher, the realization that we are developing to a competition, and not necessarily to progress science and understanding is a hard compromise to make. It is like teaching to the exam rather than ensuring the students understand material. However, because the Cybathlon tasks were designed to closely resemble real world scenarios, developing for the challenge is not too far removed from advancing the field, and I know we would revisit this work after the competition.

With the exoskeleton ready for Mark, his job was to train as much as possible. Unlike with the DRC, where we could operate the robot almost continuously, for the Cybathlon we did not want Mark to overexert himself and risk injury. We also had to finish developing software, tuning parameters, while fixing any broken hardware. In the course of the final eight weeks, we had to completely disassembled the exoskeleton and reassembled it twice, which took time away from training and development. We targeted three to four training days per week, with four hours of training per day. When Mark was not training, we were testing newly developed features and maintaining the hardware.

As with the DRC, we knew the value in recreating the tasks as close to the final ones as possible. Fortunately, the Cybathlon organization published the exact specifications of the course, so there would not be any unexpected challenges. We started training with flat ground walking and standing up and sitting down because they were the easiest tasks, and the ones that required the least amount of software development. In addition, these tasks were fundamentally critical to the success of the other tasks. At the same time that Mark was learning how to walk and balance in the exoskeleton, we were improving the walking trajectories and tuning the timing parameters.

One of the main areas for development was how to command the powered ankle, especially during the toe-off portion of the gait cycle. Our initial plan was to leverage the algorithms from our humanoid work, which would utilize compliant control at each of the joints. However, this plan was one of the developments that was tossed overboard, resulting in us controlling the actuators using position control based on predetermined trajectories. The position control is much stiffer and less accommodating to unexpected variations or changes in the ground profile.

The development of the control algorithms for the Cybathlon was significantly different from that of the DRC. For the DRC, the walking and balance algorithm had to work perfectly, where any error in stability would result in a fall. The operator controlling the robot could only provide high level commands, so all of the balance and stability had to be encoded in algorithms. Any bug or miscalculation in the algorithms due to an unexpected or untested situation could result in the robot falling. With the exoskeleton, we only need to get the walking trajectories close to the “optimal” solution, and the pilot could compensate and adapt to whatever motion the exoskeleton was providing, or not providing. For the sake of time, it was more important to lock down the trajectories early, and possibly have them be suboptimal, so that the pilot could have as much time to train with a given, and predictable, set of motions.

For each task of the Cybathlon, we worked with Mark and strategized what was the best way to complete it. For example, with the sofa task, because the seat is so low, we tried putting an extra set of handles on the crutches. For the stepping stone task, we used the provided stone spacing to preprogram the step sizes. While we felt this was slightly gaming the system, it would have been too time consuming during the competition to have Mark specifically select each step size. For opening and closing the door, we tried to find out the exact model of door handle, since European handles are generally levers whereas the American ones are generally knobs. While we tried to ensure that our solutions would work for a variety of situations, we balanced that with the competition aspect. We brainstormed several different techniques, including strings with magnets and loops. We eventually settled on affixing hooks to the base of the crutches, one to twist the handle open and one to pull the door shut. The question of descending the stairs forward or backward was debated among the team. What lead us to select backward was Mark felt more comfortable, and the swing trajectories were almost identical as ascending, except in reverse.

With about two weeks before we had to pack up, Mark was able to complete five tasks in close to the ten-minute time limit. Thinking that it was not possible for Mark to reliably speed up his performance enough to have time for the sixth task, we decided our game plan would be to skip the tilted path task at the competition, and therefore not even train for it. By not training for that task, Mark was able to focus on the five others, while the engineers would also not have to spend time developing software specific for that task.

With three days before we packed up, Mark was able to complete the same five tasks in about nine minutes. This improvement in performance resulted in the team revisiting the decision of training for the sixth task. This debate really made the project feel like a competition and not simply a research project. We still did not know how the other teams were doing, and assumed that there would be at least several able to complete all six tasks in under ten minutes. Arguments in favor of doing the sixth task were that we should try to get as many points as possible, and if there was a chance we could do all six tasks, then we should. There were two arguments against: one was that if we tried the tilted path and then did not have time for the stairs (the final and most valuable task), we might lose to a team that skipped one of the first five. The other reason was that I did not want to put pressure on Mark and risk that he feel like he let us down if he failed that task. It is the sentiment that this is an athletic competition that is highly tied to the pilot’s performance, and is what highlighted the difference between the Cybathlon and the DRC. In the end, we stuck to our initial decision and decided to skip the tilted path task.

Travel to Zurich for the team was more than just attending a competition; for several of the team members, including Mark, it was their first time in another country. We arrived at the hotel and immediately turned one of the rooms into a make shift robot workshop. We then unpacked and assembled the exoskeleton to start testing before anyone went to bed to verify that everything was working after shipment. Up until this point, Mark had always operated the exoskeleton with an overhead fall prevention system. Walking at the hotel was the first time operating without one, and we were all a little nervous, except Mark. All of the hardware survived the travel and everything was working great.

For the team, and especially Mark, the feeling at the actual competition was more excitement than nervousness. My biggest concern was that there would be a hardware problem before or during the competition, and then Mark would not be able to compete. Coming from the research world, we are generally happy if our hardware works occasionally, as long as we can get it working on film and collect some data. What helped us feel relaxed was our extensive training and consistent and repeatable performance in the lab. Our hope was to complete the five tasks in under ten minutes, just as we trained, without any real expectation on how we would place compared to the other teams.

Our two runs at the Cybathlon went just as planned. Aside from Mark almost dropping his crutch over the side of the stairs, there were no issues with Mark’s performance or the hardware. Much to our surprise, and joy, we placed second overall, just like we placed second at the DRC Finals. We crossed the finished line in the finals with 1 min 20 s left out of a total of 10 min for the run. Would this have been enough time left to complete the sixth task? It is something that we did not dwell on because we were ecstatic with second place, and could not have asked for a better showing.

Once the stress of keeping the hardware, and Mark, in working order for the Cybathlon was over, we decided to be a little more adventurous. The day after the competition, Mark walked at a few places around Zurich, which was the first time he took the exoskeleton outside and in public. While Mark was able to walk around, it did highlight how much work we have to do to improve the capacities of our powered exoskeleton to the point that they are ready to be used for the general population.

## Conclusions

Engineering championships, like the Cybathlon and the DRC can be great opportunities for researchers. As long as the tasks or challenges in the competition encourage scientific advancement, the significant effort required for the competition can be leveraged for the ongoing research. Competing in the three phases of the DRC brought a focus and intensity to our research group for the two years that we were working in it. Having a relatively long term, goal oriented project, resulted in a base of foundational software that has benefited many subsequent projects. To this day, the Atlas robot from the DRC is still our main hardware platform to develop and test our walking, balancing, manipulating, perception, and planning algorithms.

The memories of competing in the Cybathlon are different from the DRC, and it mostly relates to the fact that, there is a human athlete at the center of the competition. Participation in the Cybathlon was my most rewarding professional endeavor. The opportunity to work with Mark, our pilot, and see how our technology can offer the hope to walk again is rare for a robotics researcher. Participating in these types of competitions also brings comradery not only within the team for a more enjoyable work environment, but between the teams, for increased collaboration with other research groups. The announcement of the Cybathlon 2020 will ensure that these experiences and focused developments can continue.

Over the next several years, there is significant opportunity for improving the performance and capabilities of powered exoskeletons. The main areas for improvements are speed, balance, maneuverability, and user interface. The straight line walking speed needs to be increased to about 1.5 m/s, the point in which the pilot can keep up with an able-bodied person walking at a normal pace. Currently, none of the exoskeletons at the Cybathlon contributed actively to maintaining or assisting with balancing. Full balance control can only be achieved with at least six actuators per leg, and at a minimum, sagittal plane balance assistance is possible with Mina v2. The ability to side step and turn would increase the maneuverability of the user and result in a more capable device. Finally, as more capabilities are added, the user interface needs to be enhanced so that the cognitive load of operating the device remains minimal. It is my hope that IHMC and the other research and commercial companies working in this area can make advances in these areas over the next few years.

## Abbreviations

DRC: DARPA Robotics Challenge
IHMC: Florida Institute for Human and Machine Cognition
MIT: Massachusetts Institute of Technology
